# Ultrasound Open Platforms for Next-Generation Imaging Technique Development

**DOI:** 10.1109/TUFFC.2018.2844560

**Published:** 2018-06-06

**Authors:** Enrico Boni, Alfred C. H. Yu, Steven Freear, Jørgen Arendt Jensen, Piero Tortoli

**Affiliations:** 1 Department of Information EngineeringUniversity of Florence50139FlorenceItaly; 2 Schlegel Research Institute for Aging, University of WaterlooWaterlooONN2J 0E2Canada; 3 Department of Electrical and Computer EngineeringUniversity of WaterlooWaterlooONN2L 3G1Canada; 4 School of Electronic and Electrical EngineeringUniversity of LeedsLeedsLS2 9JTU.K.; 5 Center of Fast Ultrasound ImagingDepartment of Electrical EngineeringTechnical University of Denmark2800LyngbyDenmark

**Keywords:** Next-generation imaging technique, open platform (OP) ultrasound scanner, programmability, system architecture

## Abstract

Open platform (OP) ultrasound systems are aimed primarily at the research community. They have been at the forefront of the development of synthetic aperture, plane wave, shear wave elastography, and vector flow imaging. Such platforms are driven by a need for broad flexibility of parameters that are normally preset or fixed within clinical scanners. OP ultrasound scanners are defined to have three key features including customization of the transmit waveform, access to the prebeamformed receive data, and the ability to implement real-time imaging. In this paper, a formative discussion is given on the development of OPs from both the research community and the commercial sector. Both software- and hardware-based architectures are considered, and their specifications are compared in terms of resources and programmability. Software-based platforms capable of real-time beamforming generally make use of scalable graphics processing unit architectures, whereas a common feature of hardware-based platforms is the use of field-programmable gate array and digital signal processor devices to provide additional on-board processing capacity. OPs with extended number of channels (>256) are also discussed in relation to their role in supporting 3-D imaging technique development. With the increasing maturity of OP ultrasound scanners, the pace of advancement in ultrasound imaging algorithms is poised to be accelerated.

## I. Introduction

Ultrasound imaging has enjoyed tremendous success as a real-time imaging modality for bedside diagnostics [1]. This success is much attributed to various engineering advances such as array transducer design [2], integrated circuit (IC) development [3], [4], and digital signal processing hardware [5], [6] that have altogether enabled real-time implementation of ultrasound imaging. Thanks to these engineering advances, clinical ultrasound scanners are generally compact enough to fit within a rollable trolley or even a portable tablet device [7], [8]. Nevertheless, such hardware miniaturization effort has unnecessarily created an impediment for researchers to pursue the design of new ultrasound imaging algorithms that operate differently from standard imaging modes, because the operations of clinical ultrasound scanners cannot be readily reconfigured due to various hardware constraints and proprietary barriers imposed during the embedded system design process. Consequently, for many years, various research groups have faced difficulties in demonstrating the clinical potential of new ultrasound imaging techniques being developed in the laboratory beyond proof-of-concept simulations derived from ultrasound field computation programs [9].

To foster the development of new diagnostic ultrasound methods, it has been publicly acknowledged for nearly two decades that open platform (OP) ultrasound scanners need to be developed for use primarily by researchers [10], [11]. In response to this need, a few ultrasound scanners with add-on research interfaces have been developed by clinical system manufacturers in the early 2000s [12]–[15]. These platforms have granted researchers with access to the system’s radio frequency (RF) data acquired after delay-and-sum beamforming, and in turn, researchers may use these raw data sets to test new signal processing algorithms. However, because these platforms are essentially extended from clinical ultrasound scanners, their transmit-end pulsing sequence must follow the same scanline-based pulse-echo sensing paradigm used in clinical ultrasound imaging. Researchers cannot flexibly change these systems’ transmit operations, nor can they obtain the raw signals detected by each array channel prior to beamforming.

In recent years, ultrasound research scanners that are truly based on the OP concept are actively being developed to more effectively facilitate the practical evaluation of new ultrasound imaging methods. Some of these platforms are developed in academic laboratories [16]–[18], while others are commercial platforms [19]. The common feature of these OPs is that they offer operational programmability in terms of both the transmission (TX) and reception (RX) operations [20], [21]. Platform users, who are often researchers and engineers, may implement alternative imaging paradigms that are distinguished from the scanline-based imaging paradigm, such as synthetic aperture (SA) imaging [22], plane wave imaging [23], shearwave elastography [24], and vector flow imaging [25], [26]. The time and resources required for such implementation are seemingly less than that needed to redesign a prototype scanner from scratch.

In this paper, we present a formative discussion on the current state of the art in OP ultrasound scanner design and emerging development trends. Not only will a historical context be provided (Section II), the general architecture for different research purpose OPs will also be presented in Sections III–V. In Section VI, we shall summarize the common design attributes of existing OPs, comparatively analyze their pros and cons, and comment on the directions for next-generation OP development endeavors.

## II. Historical Review of Ultrasound Open Platforms

### A. Early Development Efforts

The development of research purpose OPs for ultrasound imaging has a long history that started before the rapid surge of the ultrasound industry in the 1990s. The first phased array system dates back to 1974, when Thurstone and von Ramm [27] developed a platform whose beamformation was entirely analog and whose operations were controlled by a PDP-11 computer. A system for SA imaging was also developed by Burckhardt *et al.* [28]. The first fully digital research systems including some of the features discussed in Section I were characterized by having a single active channel in both TX and RX. The first digital SA system emerged in [29] and [30] using an array probe. The system had a single channel in both TX and RX, and it used multiplexing for selecting the TX/RX element. It stored the received response in 32 random access memory (RAM) blocks for digital reconstruction by dedicated hardware at a frame rate of 30 Hz. The combination of analog parallel beamforming and computer control was used to make the first real-time 3-D ultrasound system [31], which could produce 8 volumes/s.

The first research system for fully digital acquisition was described by Jensen and Mathorne [32], which was used in conjunction with a BK Medical single-element rotating probe. The system could acquire fully coherent RF data for several images and was used for deconvolution of ultrasound images [33]. A similar system called Fast Echographic Multiparametric Multi-Imaging Novel Apparatus was later developed [34], while other platforms with similar features were also built to test novel real-time multigate Doppler methods [35] and coded excitation techniques [36]. The combination of digital acquisition and array probe TX was realized in the late 1990s using RX multiplexing [37]. The TX field could be emitted by up to 64 transducer elements selected by a multiplexer from 192 elements, and a single transducer element could be sampled in RX. This made it possible to acquire compound images for stationary objects and experiment with advanced beamforming, since all data were acquired coherently. A similar approach was used to investigate the limited diffraction beams [38]. Here, a plane wave could be emitted by combining all TX elements, and a single element could be sampled by an oscilloscope limiting the use to stationary objects, although very fast imaging was investigated.

### B. Array Systems With Full TX and RX Control

The first OP with real-time TX and RX control of the entire array was the Remotely Accessible Software configurable Multi-channel Ultrasound Sampling (RASMUS) system developed by Jensen *et al.* [16], [39].

**Fig. 1.**
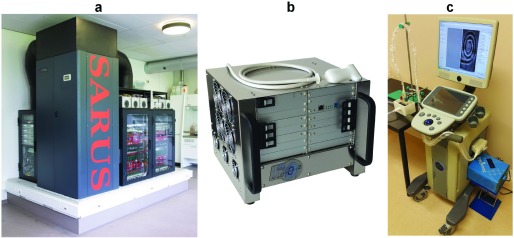
Photographs of three different ultrasound OPs. (a) SARUS developed at the Technical University of Denmark [20], [50]. (b) ULA-OP 256 developed at the University of Florence [21]. (c) Commercially available SonixTouch research scanner with channel domain data acquisition capabilities [61].

Here, arbitrary waveforms could be transmitted on up to 128 channels in parallel, and the waveforms could change from element to element and from emission to emission. Data could be sampled at 40 MHz and 12-bit resolution for 64 channels in parallel and stored in 16 GB of RAM. Two-to-one multiplexing in receive gave the ability to use 128 element probes. The generous RAM made it possible to store data for several seconds, thus capturing several heart beats. The processing was based on field-programmable gate array (FPGA) with programs written in VHDL. Real-time processing was also possible to generate an orientation image for *in vivo* acquisitions. The system was controlled over an Ethernet connection using MATLAB, which gave it great flexibility in setting up new imaging schemes with a modest amount of coding. This enabled the possibility of implementing any imaging scheme such as SA spherical [22], [40] or plane wave imaging for ultrafast frame rates [41], coded excitation [42]–[44], and spread spectrum imaging [45], [46]. The fully coherent acquisition and processing also made it possible to demonstrate *in vivo* vector flow imaging at very high frame rates [40] as well as *in vivo* transverse oscillation vector flow imaging [47]–[49]. The second generation of the Danish system called SA real-time ultrasound system (SARUS) was developed in [50], where the channel count was expanded to 1024. The SARUS system, a photo of which is shown in Fig. 1(a), can send out arbitrary coded signals on all 1024 channels and can receive simultaneously on all channels for full 3-D imaging with matrix probes. Data can be stored in the 128-GB RAM for postbeamforming, or real-time full SA beamforming can be performed using the 320 FPGAs in the system [20]. The key specifications of SARUS are listed in Table I (Column 1). It will be further described in Section V.

**TABLE I:** Main OPs Specifications

	SARUS	ULA-OP 256	UARP	SonixTouch	Verasonics (Vantage 256)
Channels	Up to 1024 Tx/Rx	Up to 256 Tx/Rx	Up to 256 Tx/Rx	128 Tx/Rx	256 Tx/Rx
Tx Voltage	Up to $200~V_{pp}$	Up to $200~V_{pp}$	Up to $200~V_{pp}$	Up to $50~V_{pp}$	3 to $190~V_{pp}$
Tx Frequency	1 to 30 MHz	1 to 20 MHz	0.5 to 15 MHz	1 to 20 MHz	0.5 to 20 MHz (standard config.)
Tx Type	Linear	Linear	5-Level	3-Level	3-Level
ADC	70 MHz @ 12 bits programmable downsampling with filtration	78 MHz @ 12 bits programmable downsampling	programmable sampling rate up to 80 MHz @ 12 bits	80 MHz @ 10 bits/40 MHz @ 12 bits	programmable sampling rate up to 62.5 MHz @ 14 bits
RAM Buffer	128 GB	80 GB	16 GB	16 GB	16 GB
Connection to PC	sixty-four 1Gb/s Ethernet links coupled through four 10Gb/s optical links	USB 3.0	PCIe 3.0	USB 2.0	PCIe 3.0

Another 128-channel system was developed by Tanter *et al.* [24] for the purpose of testing shear wave elastography methods. For this system, plane wave could be emitted in the kilohertz range for ultrafast imaging and data could be stored in the 2-MB memory for each of the channels making it possible to acquire 200–300 RF data sets. The Fraunhofer Institute developed the DiPhAS phased array system capable of real-time processing of 64 channel data [51]. Bipolar TX is performed at a 120-MHz sampling frequency and the received data are sampled at 12 bits. The system could use high-frequency probes up to 20 MHz. It could be programed to perform real-time processing for various applications. A high-frame-rate system for investigation limited diffraction beams was made by Lu *et al.* [17]. It is a full system like the RASMUS system with 128 independent channels, 40-MHz/12-bit converters used for both transmit and receive and generous RAM resources with up 512 MB per channel for deep memories for acquiring longer *in vivo* sequences of, for example, the heart. The system could not perform real-time beamforming, which had to be performed on a personal computer (PC) after acquisition.

### C. Open Platforms With Transportable Size

The OPs described in Section II-B were quite bulky and not easily transportable. This drawback was remedied by the ultrasound advanced OP (ULA-OP) system developed by Tortoli *et al.* [18], [52], which is a compact system with the capability of processing 64 channel data in real-time for a 192-element probe. This table-top system ($34 \times 23 \times 14$ cm) can send out arbitrary waveforms, real-time process the data, and can store up to 1 GB of data.

The system has been widely adopted by the ultrasound research community, and a large range of groups are using it for developing new imaging schemes and testing them out [53]. A new generation of the system, which is described in detail in Section IV, has increased the channel count to 256 and added more processing resources and RAM, while maintaining the transportability [21]. A photograph of this new system is shown in Fig. 1(b), and its hardware specifications are summarized in Table I (Column 2).

In the U.K., the ultrasound array research platform (UARP) system was made by Smith *et al.* [54]. Table I (Column 3) shows the main system specifications of UARP. This scalable system is based on 16-channel Peripheral Component Interconnect Express (PCIe) modules, each equipped with 1-GB DDR3, Stratix V FPGA. The excitation scheme is an efficient metal-oxide-semiconductor field effect transistor (MOSFET)-based design [55] and generating arbitrary sequences with harmonic control [56]. The system is racked mounted on commercial PCIe backplanes for imaging applications where large channel numbers (128–512) are required. The on-board FPGA implements a programmable 100-tap finite impulse response filter on each channel and performs signal equalization. Partially beamformed data are sent to the controlling PC, where further elaboration is done. The UARP has been used for harmonic imaging schemes [57], contrast agent studies [58] through to NDT applications [59].

Multichannel research systems have also been developed by other research groups. Lewandowski *et al.* [60] constructed a system capable of real-time graphics processing unit (GPU) processing. As well, Cheung *et al.* [61] have made an add-on tool for use with Ultrasonix research scanners. This latter platform is shown in Fig. 1(c). Its hardware specifications are summarized in Table I (Column 4).

### D. Commercial Systems for Research Purpose

In response to a workshop sponsored by the National Cancer Institute that underscored the need for research purpose ultrasound systems [10], a number of commercial research platforms have evolved spanning both digital beamformed data as well as raw multichannel data from the individual transducer elements. The single channel beamformed data option has been provided by Siemens [62], Hitachi [13], Ultrasonix [14], BK Medical [63], and Zonare [15]. All of these systems have the capability of storing the summed RF data from the beamformer, so further experimentation with back-end processing can be made. They also allow some experimentation with other imaging schemes, but companies are often reluctant to give access to all features due to the inherent safety risk from experimental TX sequences. Information about early research systems can be found in a 2006 special issue of the IEEE Transactions on Ultrasonics, Ferroelectrics, and Frequency Control [11].

Since these early developments, a number of multichannel systems have evolved in recent years. Verasonics (Kirkland, WA, USA) currently markets a widely used commercial system that offers full flexibility in TX and sampling of 256 element transducers with flexible back-end processing [see Table I (Column 5) for its main specifications]. Several of these systems can even be synchronized and this has been used to sample 1024 element matrix probes. Other similar systems have been put on the market by Ultrasonix (Richmond, BC, Canada) and US4US (Warsaw, Poland). A research purpose system was also developed by Alpinion (Seoul, South Korea), but it seems to be temporarily withdrawn from the market. Cephasonics (Santa Clara, CA, USA) has specialized in delivering systems and components for research systems, and their products can be tailored from 64 to thousands of channels for sampling individual element signals. Similar products are available as well from Lecouer Electronique (Chuelles, France).

## III. Architecture of Open Platforms: Software-Based Platforms

Since an OP ultrasound scanner should ideally allow researchers to implement any new imaging algorithm, its hardware components should be designed such that their TX operations of every array channel can be reconfigured and the data processing chain can be flexibly programed. This dogma in OP design has been practiced in a few different ways. For OP scanners that implement data processing routines through computer programming, we shall categorize them as software-based OPs to underscore the fact that their operations can be programed in a software environment using high-level programing languages. Their architecture generally consists of various functional modules as described in Sections III-A–III-D.

### A. Front-End Electronics

**Fig. 2.**
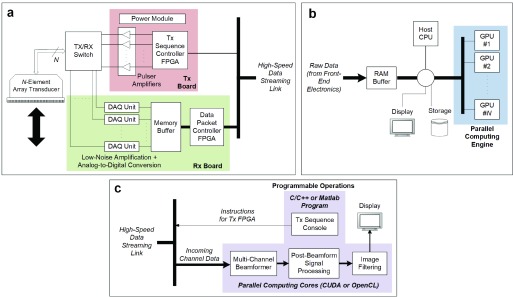
General architecture of software-based OPs with (a) FE electronics and (b) back-end computing engine. (c) TX and RX operations are generally programmable using a high-level language.

The TX operations of software-based OPs are realized using analog electronics in ways that are similar to clinical ultrasound scanners. As illustrated in Fig. 2(a), the following major TX-related hardware components can be found in software-based OPs: pulser amplifiers (for driving individual array elements), a power distribution module (for supplying the required electrical voltages), and a TX sequence controller (for setting the pulse pattern to be sent through each array element). These electronic components are generally housed within a multilayer printed circuit board (PCB), and the pulser amplifiers and power distribution module are typically implemented using commercially available IC chips [3], [4].

There are alternative approaches to the implementation of the pulser electronics to facilitate arbitrary waveform generation. These approaches generally involve the use of digital-to-analog converter with linear power amplification [64] or MOSFET-based switches [55]. Linear power amplifiers offer the broadest waveform flexibility, although this is achieved at the expense of space integration and power dissipation. In fact, they are usually packed in two channels per chip maximum, and the chip size is in the order of 1 cm^2^. Also, the linear circuits need to be biased with some current from the high-voltage rails. On the other hand, square-wave MOSFET pulsers (either three or five levels) offer less flexibility in generating the output waveform, even if special excitation methods are used [55], [56]. Yet, their power efficiency is higher than that for linear power amplifiers. As well, space integration is a plus, since the market offers ICs that integrate 16 channels, five-level pulsers in 1 cm^2^ to support arbitrary waveform generation [65].

As for the TX sequence controller, it is implemented using an FPGA as opposed to hardwired logic. On the RX side, since the processing operations of software-based OPs are carried out in the computing back-end, the corresponding analog electronics contain fewer components than those found in clinical ultrasound scanners and other types of OPs. In particular, the RX circuit board of software-based OPs only contains the following functional components: TX/RX switches, data acquisition units, an on-board RAM buffer, and a data packet controller. Note that both the multiplexer switches and data acquisition units are implemented using commercial ICs, while the data packet controller is in the form of an FPGA [61]. RF sampling rates between 40 and 80 MHz with the bit resolution ranging between 12 and 16 bits are readily achievable nowadays.

### B. Data Streaming

Unlike clinical ultrasound scanners, software-based OPs do not have a hardware beamformer, nor on-board computing devices. Instead, all the acquired channel data are fed to the computing back-end for processing. This data handling strategy necessitates the use of a high-speed data streaming link because with the concerned data volume can be rather large in size. For instance, for a software-based OP with 128 channels and operating at 40-MHz RF sampling rate (with 16 bits per sample or 2 bytes), each TX pulsing event would generate a raw data size of 1.024 MB for an axial imaging depth of 7.7 cm (assuming a speed of sound of 1540 cm/s). With 10 000 TX events every second [i.e., a pulse repetition frequency of 10 000 Hz], the raw data volume would be of 9.537 GB in size. Such a raw data volume inherently cannot be transferred in real time to the computing back-end using universal serial bus (USB) links [61]. As such, data transfer links with high bandwidth are typically deployed in software-based OPs. One representative example is to make use of multiple PCIe links, each of which has a theoretical data bandwidth of 8 GB/s (excluding overhead) for version 2.0 technology and 16 parallel lanes [19], [66]. To make use of this data transfer link, the RX hardware’s data packet controller FPGA is typically preprogrammed with a commercially available driver core that contains the necessary register transfer level (RTL) descriptions for synchronized high-speed data streaming. Also, a PCIe hardware switch is deployed to facilitate the direct streaming of data packets to back-end computing devices [66], [67].

### C. Back-End Computing Engine

The back-end computing engine of software-based OPs is responsible for executing the entire signal processing chain that regards raw channel data frames as its input. This computing engine is typically a high-end PC workstation. As shown in Fig. 2(b), during operation, incoming raw data are fed from the front-end (FE) hardware. Since this incoming data traffic is on the order of gigabyte in size every second, it is imperative for the workstation to be equipped with sufficient computing resources to handle such a large data volume. While it is possible to perform processing by leveraging the on-board central processing unit (CPU) [19], its processing capacity is fundamentally limited by the CPU’s clock speed, and thus, the processing would need to be done on a retrospective basis. To overcome this issue, GPU has been leveraged as an enabling technology to facilitate high-throughput parallel processing of raw data samples [68]. The key benefit of using GPUs is that each of these computing devices contains thousands of processor cores (more than 3000 cores with latest technology), so it is well suited for high-throughput execution of single-instruction, multiple-thread computing algorithms [69], [70]. Multiple GPU devices may be connected to the workstation to scale the OP’s computing capacity. Note that GPUs are after all graphics rendering devices. Thus, it is well possible to concurrently leverage some of the GPU resources for visualization operations.

Using GPU processing, software-based OPs have demonstrated that delay-and-sum beamforming may be readily achieved at real-time throughputs [71], [72]. Other GPU-based beamforming algorithms have also been explored, such as spatial coherence imaging [73] and minimum variance apodization [74]. Note that GPU processing is not limited to beamforming operations. Various postbeamforming signal processing operations may also be performed using the GPU, such as Doppler imaging [75] and related adaptive clutter filtering operations [76], motion estimation in elastography [77], [78], temperature mapping for therapeutic monitoring [79], as well as image filtering [80]. It is also possible to integrate different GPU processing modules to realize more advanced algorithms such as high frame-rate vector flow estimation [81] and color-encoded speckle imaging [82]. The latter has particularly been integrated with a software-based OP FE to achieve live imaging of arterial and venous flow dynamics [83].

### D. Programmability of System Operations

Since software-based OPs perform data processing operations via the back-end PC, the corresponding computer software is naturally different from that of clinical scanners. Specifically, in addition to the software-based user interface, code modules are developed to handle various system-level operations on both the TX and RX sides. As illustrated in Fig. 2(c), users are typically granted access to the software to reconfigure the TX sequence in the form of a computer program. In particular, the system manufacturer would provide a set of software-level application programming interface (API) libraries [84] that can parse a series of user-defined operational parameters programed using the C/C++ language and perform the corresponding hardware-level instructions to reprogram the TX sequence controller FPGA to execute a customized TX strategy. A similar concept may be realized using the MATLAB scripting language [19]. By adopting a high-level programing approach to redefine the system’s TX operations, research users do not need to spend time on developing low-level RTL descriptions using hardware description languages such as Verilog and VHDL to reprogram the system’s FPGAs. Instead, they can focus on imaging strategy design tasks that are more research oriented and work with a high-level programing language such as C/C++ or MATLAB that they are more likely to be familiar with.

For RX operations, research users have flexibility in implementing a variety of signal processing algorithms using high-level programing languages. If GPU-based parallel processing is to be performed, the corresponding computing kernels may be developed in the C language with appropriate syntax modifications that are aligned with a GPU vendor specific API such as compute unified device architecture (CUDA) (NVidia; Santa Clara, CA, USA) [85] or a universal API like Open Computing Language (OpenCL) [86]. These GPU computing kernels may be readily integrated into MATLAB scripting routines by compiling the corresponding source code as MATLAB executable files. Also, for parallel computing kernels that are coded using OpenCL, they can be converted into RTL instructions using high-level synthesis (HLS) tools for execution on FPGAs that are mounted as parallel computing devices on the PC motherboard [87]. Overall speaking, software-based OPs offer researchers the convenience of using C/C++ or MATLAB to prototype new signal processing methods that work with raw channel data. The savings in development time effectively serve to accelerate the pace of development for new ultrasound imaging techniques.

## IV. Architecture of Open Platforms: Hardware-Based Platforms

In contrast to software-based OPs, some research scanners realize data processing via on-board computing hardware such as FPGA, digital signal processor (DSP), and system on chip (SoC). For these latter platforms, they will be referred to as hardware-based OPs in light of their on-board processing approach. Their general system organization and programmability are described in Sections IV-A–IV-D.

### A. General System Organization

**Fig. 3.**
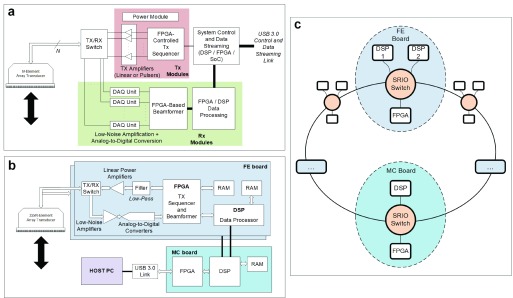
Conceptual overview of hardware-based OPs. (a) General organization of such systems. (b) Block diagram of the main hardware modules of the ULA-OP 256 system (an example of hardware-based OPs). (c) Serial RapidIO connection diagram of different ULA-OP 256 modules and their on-board computing devices.

The general architecture of hardware-based OPs is shown in Fig. 3(a). The FE electronics of such scanners [power module, pulsers, TX/RX switches, and analog-to-digital converters (ADCs)] are mostly equivalent to those of software-based systems, since in both types of OPs, the functional role of the FE circuitry is to interface the OP with the connected array probe on a channel-by-channel basis. The major difference in the hardware organization of hardware-based OPs lies in the on-board digital processing blocks that manifest as one or more FPGAs, DSPs, and SoCs. These on-board computing resources are powerful, programmable devices that are tasked to handle a cascade of signal processing operations that begin with beamforming and may also include back-end image filtering prior to display. As will be discussed in Sections IV-B–IV-D, FPGAs are often assigned to handle beamforming tasks, and they can be used either alone or in combination with DSPs to perform other signal processing tasks in real time.

Because most signal processing operations are handled by on-board computing devices, hardware-based OPs inherently do not need to send an enormous amount of raw data to the back-end PC that mainly serves as a user interface. Instead, only the beamformed RF data or baseband processed data need to be streamed from the FE electronics to the back-end PC. For the data size calculation example presented in Section III-B, the beamformed RF data traffic bandwidth is 76.294 MB/s for hardware-based OPs, and this is significantly smaller than the gigabyte-range data traffic that needs to be streamed in software-based OPs. Note that the data stream size for hardware-based OPs would be further reduced if only the demodulated or downsampled baseband data are sent to the back-end PC. Such traffic can be readily streamed in real time through the use of popular buses like the USB 3.0, which is by far less costly than PCIe links and is compatible with low-cost laptops.

One point worth noting in hardware-based OPs is that they typically house a plentiful amount of RAM to store large volumes of raw channel data that can be streamed on-demand to the back-end PC on an offline basis. For example, 80 GB of RAM has been installed on a recently developed hardware-based OP [88]. This abundant on-board memory makes it possible for researchers to acquire raw data for preliminary testing of new algorithms that work directly with channel data.

### B. Hardware Architecture

A hardware-based OP may be devised using a modular design approach to effectively facilitate the scaling of system complexity in terms of both PCB design and programmability. Representative examples of OPs making use of this design approach include the RASMUS system in Section II-B and the ultrasound array research platform (UARP) system described at the end of Section II-C. A more recent example of hardware-based OPs is the ULA-OP 256 system that is capable of independently controlling 256 probe elements [21]. As illustrated in Fig. 3(b), each module of ULA-OP 256, hereinafter identified as a FE board, hosts all the electronics needed for controlling a small number (32) of TX–RX channels, including the FE circuits, one FPGA (ARRIA V GX; Altera, San Jose, CA, USA) and two DSPs (320C6678; Texas Instruments Incorporation, Austin, TX, USA). The overall channel count of the system is scaled to 256 by replicating the FE board to integrate a total of 8 FE boards in the system hardware. In ULA-OP 256, these FE boards are inserted into a backplane that housed another board called the master control (MC) board. This latter board, which includes an FPGA and a DSP, is responsible for overseeing the data collection process of all the FE boards and interacting with the back-end PC. As well, it may be leveraged for data processing if needed. Since different boards may need to communicate with each other to complete specific processing tasks, their interconnection was carefully designed according to the Serial RapidIO (SRIO) protocol [Fig. 3(c)]. This high-speed packet-switched serial bus yields a total full-duplex link data rate of 40 Gbit/s for each board-to-board interface.

### C. Data Acquisition and On-Board Processing

In the modular design approach adopted by ULA-OP 256, each FE board during its TX operation would generate 32 independent arbitrary signals, which are boosted up to 200 V (peak to peak) by linear power amplifiers and are used to drive the respective array elements. The arbitrary waveforms are obtained according to the sigma-delta approach [64], i.e., by low-pass filtering suitable bit streams that are read from the FPGA internal memory. On the RX side, each FE board is responsible for amplifying the echoes detected from 32 array elements. The raw channel echoes are relayed to four 8-channel ultrasound FE ICs (AFE5807, Texas Instruments Incorporation), where they are amplified and are digitized at 78.125 MHz with 12-bit resolution. The digitized data streams are sent to the FPGA and are stored in a 2-GB RAM storage buffer (62.5 MB per channel). Note that the storage buffer may be extended to 10 GB (312.5 MB per channel) by leveraging the 8-GB RAM controlled by the same FE board’s two DSPs, which would be accessible through the SRIO star topology.

Rather than simply storing the raw channel echoes in the buffer, the FPGA on each FE board can be programed to perform different beamforming strategies on 32 channels. For example, it may be programed to implement, in real time, the filtered delay multiply and sum beamforming algorithm that involves elementwise data processing [89], and it has been shown to be capable of improving the contrast resolution [90]. A standard delay-and-sum beamformer may be implemented as well. In this case, the FPGA capability of working at high clock frequency (240 MHz) can be exploited to perform parallel beamforming operations. A special strategy has, in fact, been implemented [88], and it has been shown to be capable of generating multiple beamformed lines after each TX event, as required for real-time plane wave imaging [23]. After FPGA beamforming, the output data may be passed to the two on-board DSPs, each of which features eight processor cores. In the real-time plane wave imaging mode, the DSPs are leveraged to perform coherent compounding of RF data obtained by transmitting plane waves at multiple steering angles. The DSPs may also demodulate the RF data into quadrature channels, and then perform low-pass filtering and down sampling to derive the corresponding baseband data.

Since the processed data from each FE board are only pertinent to 32 channels, such intermediate data need to be further processed together with the output from other FE boards in order to derive the final beamformed data samples (or baseband data) for all channels. This integrative processing task is handled by the MC board through its DSP unit. During operation, each FE board’s processor output is sent to the MC board through the ring topology, and then the MC board’s DSP would correspondingly sum the intermediate data samples from different FE boards to obtain the final beamformed (or baseband) data sample for each pixel position in the image grid. Additional postprocessing (such as data regularization and noise filtering) may be carried out on the MC board’s DSP as required. The final processed data set may be stored on a 4-GB RAM buffer present on the MC board’s DSP, or they can be directly streamed to the back-end PC (in which case, the DSP RAM would just act as a first-in-first-out memory buffer to smoothen the streaming process).

One salient point to be noted about hardware-based OPs is that their use of multiple FPGAs and DSPs makes possible the real-time on-board implementation of novel methods that demand high processing power. As said earlier, plane wave compounding may be readily achieved by properly sharing beamforming and compounding operations between, respectively, the FE board’s FPGA and DSPs. Another example of task sharing is the multiline transmit technique [91], in which the FPGA is assigned to beamform the channel echoes along the directions of simultaneously transmitted multiple focused beams, while the DSPs are leveraged to process the beamformed data to produce cardiac images at high frame rates for tissue Doppler estimation [92]. A further example is multiline, multigate vector Doppler measurements, whereby eight pairs of RF lines are simultaneously beamformed by the FPGA and Doppler processing is carried out by the MC board’s DSP [93]. Note that for processing methods that work with beamformed data, such as coded imaging [94] and coded spectral Doppler measurements [95], the computational load of the related matched filtering operations may be carried out by the FE board’s DSPs. In contrast, the MC board’s DSP may be exploited to supervise the choice of optimal subarrays out of a linear array probe and to properly process the related echo data according to an original vector Doppler approach. Such concept has been demonstrated in a clinical study [96].

### D. Programmability of System Operations

Similar to software-based OPs, the TX and RX operations of hardware-based OPs may be programed by the user. For instance, in the ULA-OP 256 system, the TX sequence may be defined through high-level text scripting in the same way as described in Section III-D. For RX beamforming, the user can configure the system by means of text files. Such files define all the general parameters of the RX beamforming strategy (number of scan-lines, geometrical definition of scan-lines, RX focusing type, apodization type, etc.). Also, depending on the desired configuration, the beamforming delays and apodization coefficients can be either calculated by the run-time software or uploaded from binary files generated by means of, e.g., MATLAB scripts that are provided with the system software package. The latter solution is adopted when the RX strategy involves nonstandard dynamic focusing beamforming. In both cases, the run-time software translates the calculated coefficients into bitstreams that are stored in the beamforming FPGA’s local memory. The correct set of coefficients is then selected, for each pulse repetition interval (PRI), by the on-board sequencer.

For RX data processing, the user can configure real-time code modules that are provided within the DSP firmware package. Again, the configuration of these prebuilt modules is described by text files that define, for each PRI, the data to be elaborated and the parameters related to the instantiated module. The run-time software activates one or more DSP cores in each FE board and configures them to process the data as requested by the user. Real-time operations are scheduled and directed by the MC board’s DSP. The processing results are usually streamed to the PC, where real-time display is performed. Configuration of the display modules is described by means of text files, which define the relevant display features. Note that since researchers are granted access to the run-time software’s C++ source code, they may readily modify this code to develop their own C/C++ application. For example, as demonstrated earlier [97], it is possible to extract the I/Q demodulated data from ULA-OP and integrate them with system programming libraries to perform 3-D compounded imaging in elastography studies [53].

## V. Open Platforms With Extended Number of Channels

The investigation of 3-D imaging and advanced beamforming necessitates the development of research systems with a very high channel count (>256 channels). These expanded platforms have a number of design features that are found in software- and hardware-based OPs as described in Sections IV-A–IV-D. Two categories of OPs with extended channel count have been developed by a few academic laboratories, as described in the following.

### A. Standalone Systems

The first OP with more than 256 channels is the SARUS scanner developed by Jensen *et al.* [20], [50]. As shown in Fig. 1(a), this platform is a standalone system, and it comprises 1024 independent TX and RX channels distributed over six transducer plugs. Signals with any delay, apodization, and waveform can be transmitted at a 70-MHz sampling frequency with a 12-bit resolution on each channel. The parameters can be changed from element to element and from emission to emission for full flexibility. All received data can also be sampled at 70 MHz using 12 bits and stored in the 128-GB RAM. The data can be processed in real time generating more than 100 beamformed lines in parallel for each emission from 256 channels. This can give real-time SA imaging at 30 frames/s and is sufficient to generate a real-time 3-D images. More advanced beamforming is relegated to postprocessing in cluster computers. The data storage speed is therefore important, and the system uses 64 1-Gb/s Ethernet links coupled through four 10-Gb/s optical links to a storage cluster. Currently, around 60–100 MB of data can be stored per second. All 1024 channels can be used simultaneously or the system can be split into four independent system, which can be used at the same time on four experiments.

**Fig. 4.**
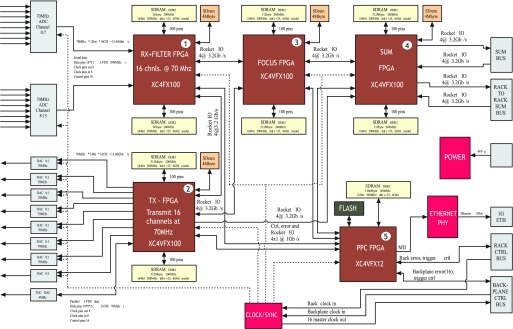
Block diagram of the FE board in the SARUS system. It houses five Xilinx FPGAs, each of which is connected to synchronous dynamic RAM. The full SARUS system consists of 64 of these boards (from [20]).

The SARUS system is controlled through commands over the network in parallel to the 64 FE boards, each of which is responsible for handling 16 TX and 16 RX channels. A Virtex-4 FPGA with a PowerPC running Linux controls the other four FPGAs on each board for controlling the TX, RX, beamforming, and summation as shown in Fig. 4. The server written in C is interfaced to MATLAB through a C communication interface, so that the commands written in MATLAB are transmitted and executed on all the boards in parallel. The MATLAB interface allows a high-abstraction level similar to the Field II simulation program [9], [98], which makes it possible to write any imaging schemes in a few lines of codes. The system is therefore remotely controllable from any location, and the resulting beamformed images can also be displayed at any location. The underlying code is roughly 960 000 lines of VHDL code, 37 000 lines of XML code, and around 91 000 lines of C code.

A standard file format has also been developed for the system, and the server automatically stores all data for a scan using just one command. The format uniquely defines the scan sequence acquired, which then can be reconstructed from the files. This makes it possible to simulate any sequence with a general program using Field II, and code has also been written to predict the emitted pressure and the corresponding intensities [99]. The measurement system can also be simulated without the actual hardware, which makes rapid prototyping possible with an indication of compliance with FDA rules before conducting measurements. The setup has been shown to be efficient in implementing all types of imaging schemes such as plane wave imaging for anatomic and flow imaging [100], SA flow imaging [101], 3-D volumetric vector flow imaging [102], [103], and a number of smaller clinical trials on volunteers have been conducted.

### B. Composite Platforms via Multisystem Synchronization

Since most available OPs are limited to control no more than 256 probe elements, a possible extension of such channel count may be achieved by the use of multiplexers interposed between the scanner and the probe. For instance, as demonstrated by the Fraunhofer Institute for Biomedical Engineering [104], it is possible to control a 1024-element 2-D array transducer through a 256-channel DiPhAS scanner. This approach, nevertheless, limits the number of array elements that can be simultaneously used, since the system electronics can only cover fewer channels than the number of array elements available. One viable alternative is to connect together more systems in attempt to control all array elements concurrently. Yet, such a composite platform assembly strategy unavoidably brings synchronization issues, since forcing different discrete systems to run on the same clock is not trivial.

The Verasonics Vantage systems (Verasonics, Kirkland, WA, USA) can be equipped with an external synchronization module that provides the needed signals to simultaneously control up to eight systems (2048 channels). One Vantage system, labeled as master, provides the logic signals to the external module, which replicates and synchronously distributes them to all the slave systems. Similarly, ULA-OP 256 [21] was designed with embedded synchronization capabilities. One master system can directly feed up to four slave systems with proper acquisition clock and synchronization signals. Each slave system can in turn feed four additional slaves. Thus, with a single level of synchronization, a combined platform (five systems) controlling up to 1280 channels can be obtained, while, in principle, with two synchronization levels, a total of 5376 channels could be controlled.

A few different applications have been so far developed through the use of such composite, multisystem strategy. For example, two synchronized ULA-OP 256 scanners are currently used at the King’s College (London, U.K.) to simultaneously control multiple ultrasound probes within the frame of the iFIND Project [105]. Elsewhere, Provost *et al.* [106], [107] have synchronized four Aixplorer systems (Supersonic Imagine, Aix-en-Provence, France) to drive a $32 \times 32$ piezocomposite matrix array centered at 3 MHz with 50% 3-dB bandwidth and 0.3 mm pitch (Vermon, Tours, France). The resulting system had 1024 channels TX capability and 512 simultaneous channels RX capability. The receiving path was multiplexed to address the full matrix. The system was used to assess the feasibility of 3-D ultrafast imaging and Doppler *in vivo*. In [108], four Verasonics Vantage systems were combined to experimentally test different 4-D ultrasound imaging modalities based on the use of 2-D sparse array elements. The selection of the active elements from the aforementioned 1024-element (Vermont) matrix probe was, here, based on a simulated annealing algorithm considering multidepth beam patterns as energy functions [109].

## VI. Discussion

### A. General Comparison of Open Platforms

To foster innovations in ultrasound imaging algorithms, it is important for an OP ultrasound scanner to possess three technical attributes.

1) Its TX operations should be programmable on a per-channel basis.
2) Prebeamform RX data should be accessible over all transducer channels, and a significant amount of RAM is available to store the data samples from multibeat acquisition.
3) Abundant computing resources should be included to allow real-time implementation of new data processing methods.

These attributes are nowadays included in either hardware-and software-based OPs. Both types of systems are usually supplied with high-level libraries to control the system operations, so the user (i.e., an ultrasound researcher) does not need to know all the implementation details. Imaging schemes can, thus, be implemented on a high level with only knowledge about the imaging scheme and not the actual hardware-level operations.

In terms of the ease of programing, software-based systems are, perhaps, easier for researchers to work with since their user-level programing environment does not require knowledge of low-level hardware description languages. For these software-based OPs, various system control operations and data processing routines are handled using high-level programming languages (C/C++ and MATLAB) and well-established parallel computing APIs (CUDA and OpenCL). The caveat in working with these platforms is that the design of parallel processing kernels still requires some level of craftsmanship in order to optimize their processing performance. Also, although GPU is the predominant parallel computing hardware used in software-based OPs, this type of computing device tends to be less power-efficient than other computing devices such as FPGAs [87].

For hardware-based OPs, the developer must be proficient in both low-level programming languages (Verilog and VHDL) to set the RTL descriptions for FPGAs and high-level languages to program the routines to be executed on DSPs. Also, since the on-board computing resources may be distributed between different hardware modules, it is imperative for the developer to have a working knowledge of the system architecture. Note that there is an emerging trend to apply HLS tools to FPGA programming [87], so in the future high-level parallel computing APIs like OpenCL may be applied to program the processing operations of hardware-based OPs. Accordingly, all operational details may be defined via high-level programming, and the researcher does not need to develop mastery of the hardware electronics in order to program on a level comparable to simulation tools like, e.g., Field II.

The key benefit of hardware-based OPs is that they are well suited for real-time applications. As aforementioned, by transmitting RF beamformed or demodulated data, which is possible in these platforms, the amount of data to be transferred decreases considerably, thus reducing the data transfer issue. In contrast, software-based OPs are generally more oriented to retrospective applications since, to reduce overhead effects, the raw RF data are typically transmitted in batches (not frame by frame), and this transfer is slower than parallel processing by GPUs. Nevertheless, recently it has been demonstrated that the software-based OP developed in Warsaw [66], [67] can be modified to make it suitable for real-time color-encoded speckle imaging of arterial and venous flow dynamics [83].

On the topic of RF data access, one important feature shared by different types of ultrasound OPs is that they possess tens and hundreds of gigabytes of RAM to store full RF data frames over multiple heart beats. Such raw data storage capacity makes it possible for researchers to conduct *in vivo* studies with OPs by acquiring multibeat *in vivo* data [110] and storing these data sets for offline processing. No restrictions are then enforced on the complexity of the processing, and the image videos can later be evaluated by medical doctors for multiple patients in double blinded trials as described in [111].

### B. Future Trends of Open Platforms

The demand for more advanced OPs with an extended number of channels is poised to grow, as there is a general trend at the cutting edge of transducer design toward a greater number of elements with 2-D transducer array configurations to offer more flexibility in terms of TX beamforming (e.g., elevation focus and 3-D beam profiles). At present, only one standalone high-channel-count OP has been built (Section V-A), and composite platforms assembled from multisystem synchronization (Section V-B) are merely stop-gap solutions. To develop such high-channel-count platforms, it is essential to overcome the technical challenge of routing a large number of high-speed channels on the PCB with matched length lines. A potential workaround is to embed the data clock into the same serial stream (i.e., similar to PCIe data streaming technology) and to concurrently make use of a standardized serial interface (e.g., JESD204b; Texas Instruments Incorporation) for facilitating phase alignment between multiple ADC IC chips and the data packet controller FPGA. This newer serial standard is already gaining popularity in electronics that make use of ADCs with higher channel counts, so it is well possible to be adopted in next-generation OP systems.

It should be mentioned that in designing high-channel-count OPs, the interconnection between individual channels of the 2-D matrix array and the OP electronics (including the cabling and related analog wiring) is itself an engineering challenge that needs to be attended to, unless FE microbeamforming circuitry is included within the 2-D transducer housing. To reduce such wiring complexity, a few solutions can potentially be adopted, such as making use of sparse 2-D array designs [112], transducers that incorporate channel multiplexing schemes [113], and 2-D arrays with top-orthogonal-to-bottom-electrode (TOBE) configurations [114]–[116]. From an OP development standpoint, the realization of these solutions will require customized connector boards to be developed, while the overall channel count may be reduced to typical values available in the existing OPs. Note that the merit of using customized transducers with channel multiplexing schemes has already been demonstrated in the context of SA imaging [117], [118]. Also, TOBE 2-D arrays have been shown to be useful in devising row–column imaging schemes [119].

Another noteworthy trend related to OP development is the way in how system design partitioning is achieved in OPs (or where along the data path are computations performed on various processing devices). While GPUs may handle the entire cascade of signal processing operations that range from beamforming to back-end image filtering (Section III-C), such tasks may also be handled by the integrative use of FPGAs and DSPs (Section IV-C). In the future, as more convoluted imaging algorithms are being developed (e.g., computational imaging based on solution to inverse problems), it would be worthwhile to pursue a hardware–software hybrid computation approach that combines the strengths of GPU, FPGA, and DSP to implement these algorithms in real time. Note that the strategy for partitioning processing tasks among different computing devices is after all influenced by concurrent advances in the computing hardware technology. For instance, FPGAs are seeing a growing trend on the incorporation of hard processor systems within the FPGA floorplan, and it will allow greater end-user control of the FPGA’s computing resources without requiring new complex FPGA instructions (which not all ultrasound researchers have the skills to work with). Also, the processing throughput and the number of computing cores available in DSPs and GPUs are continuing to increase every day. These hardware advances altogether offer a high level of flexibility in executing different tactics on process load distribution within an ultrasound OP. In turn, system design partitioning will likely become a significant engineering topic of interest for real-time realization of the next-generation ultrasound imaging methods.

## VII. Conclusion

Thanks to the increasing maturity of OP ultrasound scanners, the research community is now entering another golden age where researchers are actively proposing a variety of new imaging methods and algorithms that are tested through hardware implementations and are backed by relevant experimental results derived from these implementations. Yet, it should be emphasized that the development endeavors in OP scanners are by no means complete and are still ongoing. Rapid progress in electronics and computer science is driving the next wave of OP development with high-speed, small-size ICs for both acquisition and processing, a significant amount of RAM resources as well as high-level programming of sophisticated TX–RX strategies. It is well anticipated that the performance of upcoming OPs will further increase in terms of processing power, flexibility, and ease of programming. In turn, these next-generation OPs will undoubtedly accelerate the pace of advancement in ultrasound imaging technology, thereby bestowing this versatile imaging modality with additional advantages over other competing modalities that lack equivalent research tools.
